# Experimental Investigation of the Spall Propagation Mechanism in Bearing Raceways

**DOI:** 10.3390/ma16010068

**Published:** 2022-12-21

**Authors:** Ravit Ohana, Renata Klein, Roni Shneck, Jacob Bortman

**Affiliations:** 1 PHM Laboratory, Department of Mechanical Engineering, Ben-Gurion University of the Negev, P.O. Box 653, Beer-Sheva 8410501, Israel; 2 R. K. Diagnostics, Gilon, P.O. Box 101, D. N. Misgav 2010300, Israel; 3 Department of Material Engineering, Ben-Gurion University of the Negev, P.O. Box 653, Beer-Sheva 8410501, Israel

**Keywords:** rolling element bearings, spall propagation, crack detection, fatigue crack growth, dynamic model, finite element

## Abstract

This article investigates the spall propagation mechanism for ball bearing raceways by focusing on an experimental investigation of cracks that evolve in the vicinity of the spall edge. Understanding the spall propagation mechanism is an important step towards developing a physics-based prognostic tool for ball bearings. This research reflects an investigation of different spall sizes that propagate naturally both in laboratory experiments and in the field. By using a combined model of a rigid body dynamic model and a finite element model that simulates the rolling element–spall edge interaction, our results shed light on the material behavior (displacements, strains, and stresses) that creates an environment for crack formation and propagation. With the support of the experimental results and the rolling element–spall edge interaction model results, three stages of the mechanism that control fragment release from the raceway were identified. In Stage one, sub-surface cracks appear underneath the spall trailing edge. In Stage two, cracks appear in front of the trailing edge of the spall and, in Stage three, the cracks propagate until a fragment is released from the raceway. These stages were observed in all the tested bearings. In addition, other phenomena that affect the propagation of the cracks and the geometry of the fragment were observed, such as blistering and plastic deformation. We include an explanation of what determines the shape of the fragments.

## 1. Introduction

This research is focused on the mechanism of spall propagation in ball bearings—the most common failure mechanism in rolling-element (RE) bearings. Despite the importance of identifying spall severity in bearings, the spall propagation process is still not clear. Understanding the spall propagation mechanism will allow physics-based prognostics, and permit safer and lower-cost maintenance.

**Figure 1:**
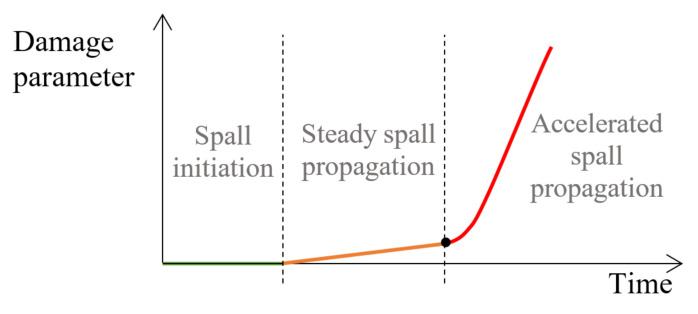
Typical failure process of a spall propagation.

Spall formation is caused by rolling contact fatigue (RCF). This is a microscopic mechanism during which micro-cracks can propagate in two different ways: (1) near the surface, originated pitting [1,2] and (2) sub-surface, originated spalling, until metallic flakes are released from the surface of the bearing raceways and/or the REs [3,4,5,6,7]. Sub-surface cracks mostly generate at stress concentration sites such as non-metallic inclusions, which causing “butterfly wings” in the vicinity of inclusions [8,9,10]. Improper installation of a bearing, or a lack of lubrication, along with the operating conditions (load, speed, and temperature) can also cause for pitting and spall formation which affect the system vibration [11,12,13,14] and eventually affect the fatigue lifetime of the bearing [15]. The spall evolution in the raceways is divided into three stages as shown in Figure 1: (1) initiation of the spall by the RCF mechanism, (2) steady spall propagation, and (3) accelerated spall propagation until final failure. After crossing the transition between the second and third stages, the deterioration of the bearing is uncontrollable and eventually will lead to catastrophic failure. Several studies have proposed methods to estimate the bearing lifetime under RCF [15,16,17,18,19,20,21,22,23]. Fracture mechanics approach has been used to model the RCF [24,25,26,27,28,29]. In addition, models have been developed to investigate the microstructure and the stochastic nature of RCF [30,31,32,33]. However, it is to be emphasized that even after the first spall formation, the bearings might be fully operational for additional millions of cycles. Therefore, it is important to investigate the damage evolution mechanisms that control the steady spall propagation stage and to identify the state of the bearing before it reaches the rapid spall propagation. Even though the spall propagation process and what affects it have been widely studied, it is still not fully understood. Several studies attempted to quantify the spall growth mechanism, such as Arakere et al. [34] and Branch et al. [35,36], who presented a 3D elastic-plastic finite element (FE) model to investigate the stress and strain fields in the neighborhood of the spall’s edge induced by the RE’s impact onto the spall’s trailing edge. The simulation results of the FE model are supported by experimental results. Arakere et al. [34] indicated that the stress field around the spall edge confirms extensive yielding and spreading of the spall, first axially (across the width of the raceway), until the RE descends into a spall. Branch et al. [35,36] found that tensile residual hoop stress appears on the spall’s edge after the RE–spall impact, which encourages fatigue crack initiation and growth. Gazizulin et al. [37] suggested a simpler quasi-static 2D plane-strain FE model for the interaction between the RE and spall edge. The simulation results are in a good agreement with those obtained in [34,36].

In the present work, we present metallographic observations of spalls from endurance tests and actual service. In particular, a follow-up of a propagating spall by scanning electron microscopy (SEM) and micro-computerized tomography (CT) during a long endurance test is described. The effect of the RE impacts on the spall edge, which eventually leads to spall propagation, was investigated by a finite element (FE) model. The displacement, stress, and strain fields at the spall edge area were examined theoretically using the FE model. This model uses results, such as the impact location between the RE and the spall edge, and the contact force from a rigid body dynamic model as an input, to simulate the impact between the RE and the edge of the spall. This methodology was validated in [37].

This article is organized as follows. Section 2 presents an RE–spall edge interaction model that integrates a rigid body dynamic model and an FE model to investigate spall edge behavior and to compare it with the experimental observations. Section 3 presents the experimental setup and results.

## 2. Model for the RE–Spall Edge Interaction

A RE–spall edge interaction model was used to explain the phenomena in the spall propagation process. Particles that are released from the spall edge of the raceway lead to propagation of the spall. According to previous studies, the spall area undergoes changes in the material, but there is still a lack of understanding of the way in which fragments release and the spall propagates. To better perceive the spall propagation process, a FE model was developed [32]. To keep the current paper self-contained, a brief explanation of the FE model is described. This section presents different physics-based models to examine the mechanism that governs the particle release and spall propagation. A rigid body dynamic model is used to evaluate the impact location between the RE and the spall edge and the contact force. These loads are used as boundary conditions for the FE model, representing the RE–spall interaction.

### 2.1. Rigid Body Dynamic Model

**Figure 2:**
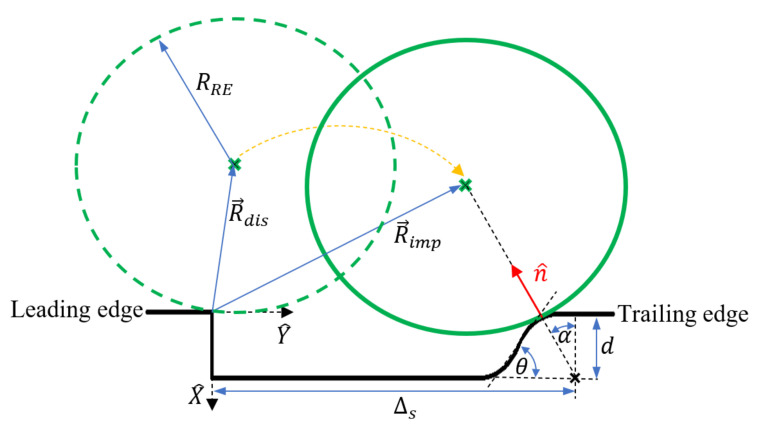
Geometric parameters definition at the moment of the RE disconnect from the raceways and impact on the trailing edge.

For a description of the interaction between the RE and the spall edge, a validated rigid body dynamic model [33] was used to calculate the impact force between the RE and spall trailing edge and the RE–spall impact location. The dynamic model developed by Kogan et al. [33] was geometrically modified by Gazizulin et al. [32], from the sharp edge of the trailing edge of the spall to an oblique edge to represent a more realistic spall edge. It estimates the first RE–spall impact location, $\left(x_{imp},y_{imp}\right)$, (Equation (1)), referring to a Cartesian coordinate system $\left(\hat{X},\hat{Y}\right)$ centered at the leading edge (see Figure 2 where all geometric parameters are shown). It also estimates the normal contact load, $F_{n}$, between the RE and spall trailing edge during their interaction (Equation (2)).

(1) $$\left\{\begin{array}{c} \begin{array}{c} \left(x_{imp},\;y_{imp}\right)=\left(R_{RE}cos\alpha -h,\;R_{imp_{y}}+R_{RE}sin\alpha \right)\\ h=\left|R_{{imp}_{x}}\right| \end{array}\\ cos\alpha =\frac{h+d}{R_{RE}+d} \end{array}\right\}$$

(2) $$F_{n}=k{\left(R_{RE}-\left(n_{o}-n_{RE}\right)\right)}^{n}\left(1+\frac{3\left(1-e^{2}\right)\left({\dot{n}}_{RE}-{\dot{n}}_{o}\right)}{4{\dot{\delta }}^{\left(-\right)}}\right)$$

where $R_{RE}$ is the RE radius and $R_{imp}$ is the RE center at the moment of impact, α is the angle between the impact direction and the $\left(-\hat{X}\right)$ direction as shown in Figure 2, k is the contact stiffness between the RE and the outer raceway, e is the coefficient of restitution, $n_{o}$ and $n_{RE}$ are the ring and RE displacements in the direction of $\hat{n}$, respectively, d is the spall depth, δ is the initial deflaction of the RE into the raceway, and ${\dot{\delta }}^{\left(-\right)}$ is the collision speed that is given by:

(3) $${\dot{\delta }}^{\left(-\right)}={\dot{R}}_{imp}\cdot \hat{n}.$$

The values of the parameters: $n_{o},n_{RE},{\dot{n}}_{o},{\dot{n}}_{RE}$ at the instant of impact are given by:

(4) $$\left\{\begin{array}{c} n_{RE}=-R_{RE}\\ \begin{array}{c} {\dot{n}}_{RE}={\dot{\delta }}^{\left(-\right)}\\ \begin{array}{c} n_{o}=0\\ {\dot{n}}_{o}=0 \end{array} \end{array} \end{array}\right\}.$$

Assuming a constant RE acceleration at the instant of impact, the velocity of the RE at impact ${\dot{R}}_{imp}$ is given by:

(5) $$\left\{\begin{array}{c} \begin{array}{c} {\dot{R}}_{imp}={\dot{R}}_{dis}+\ddot{R}t_{imp}\;\\ \begin{array}{c} R_{dis}=\left(\delta -R_{RE},\;\sqrt{2\delta R_{RE}}\right)\\ {\dot{R}}_{dis}=\frac{\omega_{c}D_{p}}{2}\left(\sqrt{\frac{2\delta }{R_{RE}}},\;1\right) \end{array} \end{array}\\ \ddot{R}=\left(g+\frac{D_{p}}{2}\omega_{c}^{2},\;0\right) \end{array}\right\}$$

where ${\dot{R}}_{dis}$ is the velocity of the RE at the moment of disconnection from the raceway, $\ddot{R}$ is the acceleration of the RE center at the moment of impact, $t_{imp}$ is the time when the RE enters into the spall until it exits from the spall, $\omega_{c}$ is the constant cage speed, and $D_{p}$ is the pitch diameter. A more detailed description of the model formulation can be found in [37,38].

### 2.2. FE Model

A quasi-static 2D plane-strain FE model was constructed with an ABAQUS/standard solver to describe the interaction between the RE and spall edge. The model was validated based on the work in [35,36]. Additional details about choosing a solver and model validation can be found in [37]. The model assumption is that the size of the defect is relatively small: the RE does not hit the bottom of the spall, i.e., the RE hovers over the spall, and only one RE is in interaction with the fault at a time.

**Figure 3:**
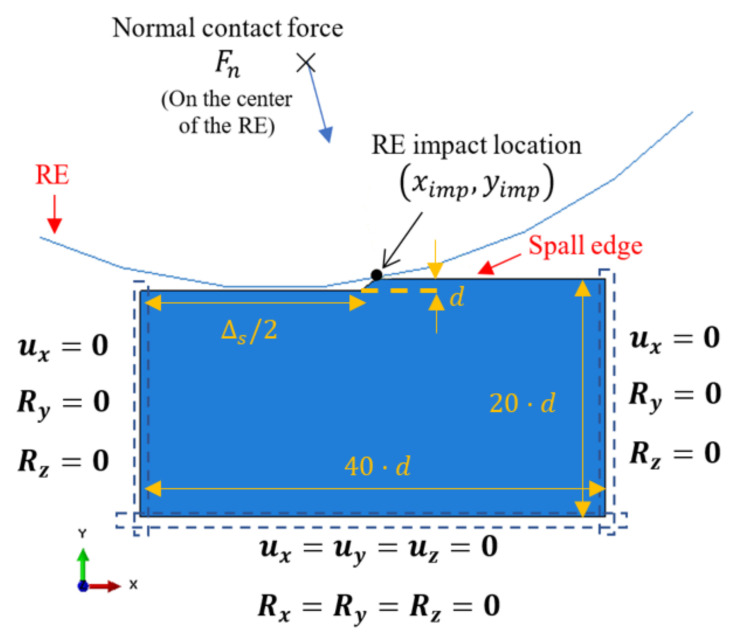
Boundary conditions and geometric parameters of the FE model. The parameters values are listed in Table 1.

The model includes two objects (Figure 3): (1) an RE, which is modeled as a rigid surface with a radius of $R_{RE}$, and (2) the spall edge. The spall edge is modeled as an elastic–plastic, homogeneous material with anisotropic strain hardening. The contact between the two objects is assumed to be frictionless. Symmetric boundary constraints (XSYM) were applied to the right and left vertical edges of the spall object (displacement constraints $u_{x}=0$ and rotational constraints $R_{y}=R_{z}=0$), and the bottom edge was fixed (Figure 3). The boundary conditions have a negligible effect on the contact problem according to Saint-Venant’s principle. The normal contact load, $F_{n}$, calculated in Equation (2), was applied to the center of the RE instance repeatedly at the RE–spall impact location, $(x_{imp},y_{imp}$), (Equation (1)). The material properties of the spall edge object are those of M50 steel [36]. Several spall sizes were simulated to examine the material behavior at the vicinity of the spall. The stress and the strain fields showed similar qualitative results. Therefore, only the results of one simulation are presented. The FE model parameters are presented in Table 1 and in Figure 3.

**Figure 4:**
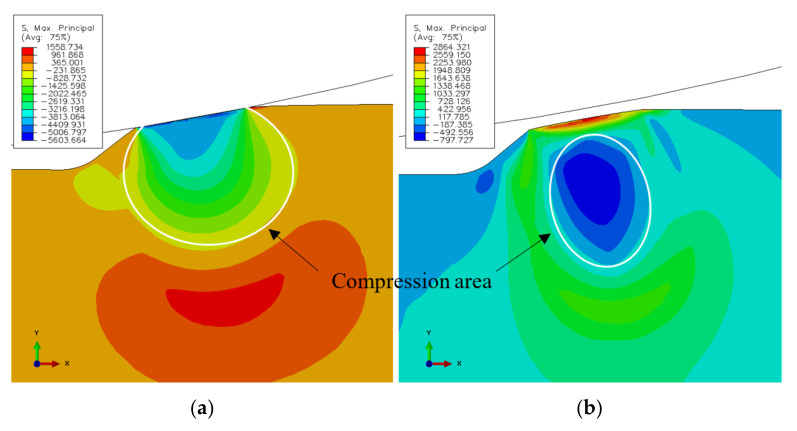
Maximum principal stress at the spall edge at the (**a**) loading and (**b**) unloading of the RE-spall interaction.

**Figure 5:**
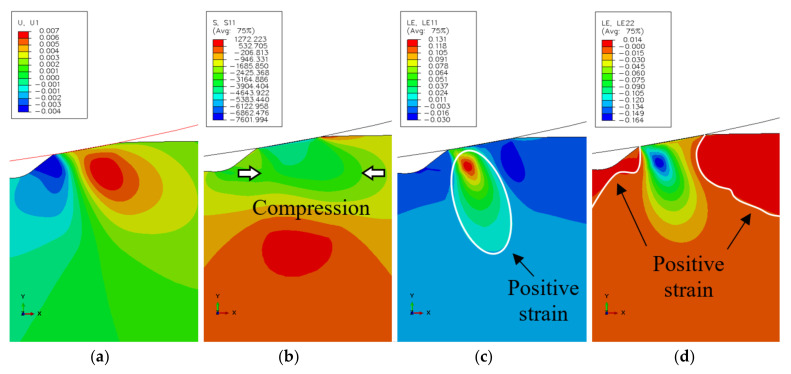
Simulation results of the (**a**) displacements in the horizontal direction x, (**b**) the stress $\sigma_{xx}$, (**c**) the strain $\varepsilon_{xx}$ and (**d**) the strain $\varepsilon_{yy}$ at the maximum normal contact load, $F_{n}$.

**Figure 6:**
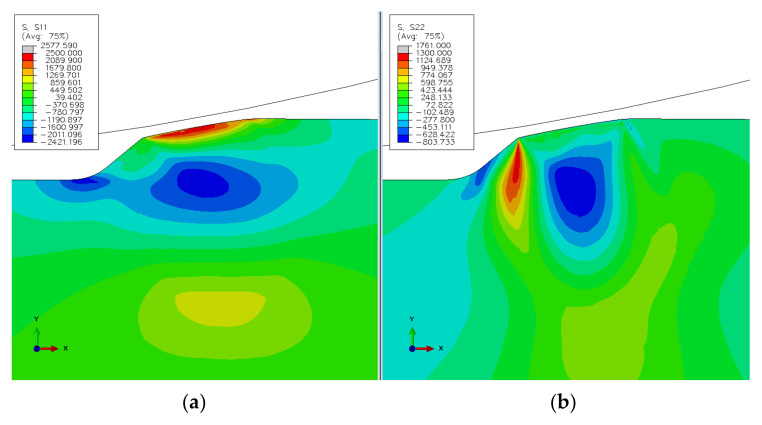
Simulated residual stresses (**a**) in the horizontal direction x, and (**b**) in the vertical direction y, in the unloading stage.

The simulated stress state at the spall edge upon loading and unloading of the RE–spall is presented in Figure 4a,b, respectively. In Figure 4a, where the normal contact load, $F_{n}$, reaches its maximum value, the spall edge is under compression stresses. Under these conditions, cracks are unable to initiate and propagate within the spall edge. However, due to the plastic deformation at the loading stage, in the unloading stage, where the normal contact load is reduced to zero, the stresses in some areas of the spall edge turn to tension. Thus, repeating the loading and unloading can lead to the appearance of cracks (Figure 4b). There is still a large area which is under compression stress. Understanding the spall mechanism requires understanding the behavior of displacements, and strain and stress fields. These are presented at the loading stage in Figure 5. The horizontal displacement (Figure 5a) is divided into an area with displacements in the positive x direction and adjacent area with displacements in the negative direction. However, the surrounding material disabled the movement, and a compressive stress appeared in the spall edge (Figure 5b). The strain fields in the horizontal and vertical directions during the loading stage, are presented in Figure 5c,d, respectively. An area with horizontal positive strain appears in the center of the spall edge (Figure 5c), and an area with vertical positive strain appears on the edges of the spall (Figure 5d). This implies the possibility of vertical crack propagation on the trailing edge of the spall and horizontal cracks from the sub-surface of the spall, despite the existence of the compression stresses. The residual stresses in the horizontal and vertical direction, after the unloading stage, are presented in Figure 6. In the horizontal direction (Figure 6a), tensile stresses are obtained on top of the trailing edge, and compression stresses are obtained deeper in the material. During the loading stage, the deeper (blue) area is subjected to tensile stresses, causing plastic deformation in the horizontal direction. After the unloading stage, the surrounding areas, which were subjected to elastic deformation, are able to return to the initial position, therefore, causing pressure on the area with the plastic deformation. Conversely, because the plastic area causes tensile stresses in the surrounding areas, residual tensile stresses evolve close to the spall trailing edge surface. The reason that there are no high tensile stresses that develop below the compression area (blue area) is that the stresses are divided over a larger area. The residual tensile stresses that evolve in the trailing edge can cause crack propagation across the trailing edge. This result agrees with the crack locations seen around the spall edge (see Section 3.2). The maximum residual tensile stresses in the vertical direction are obtained in the region of the free edge of the spall, as can be seen in Figure 6b. As explained earlier, this phenomenon can explain the progression of sub-surface cracks in the horizontal direction, which start from the free edge, as observed in the experimental results (see Section 3.2).

## 3. Experimental Setup and Results

### 3.1. Methodology

**Figure 7:**
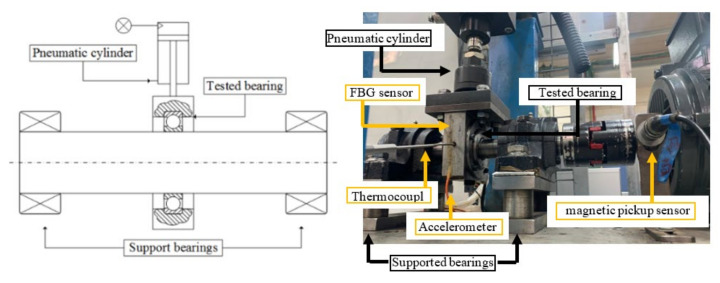
A schematic drawing and a picture of the test rig.

**Figure 8:**
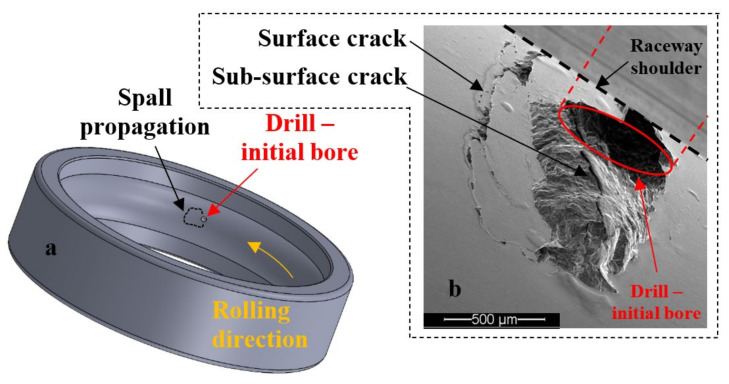
(**a**) Schematic drawing of the outer raceway with the initial bore. (**b**) Spall generated from the initial bore.

**Figure 9:**
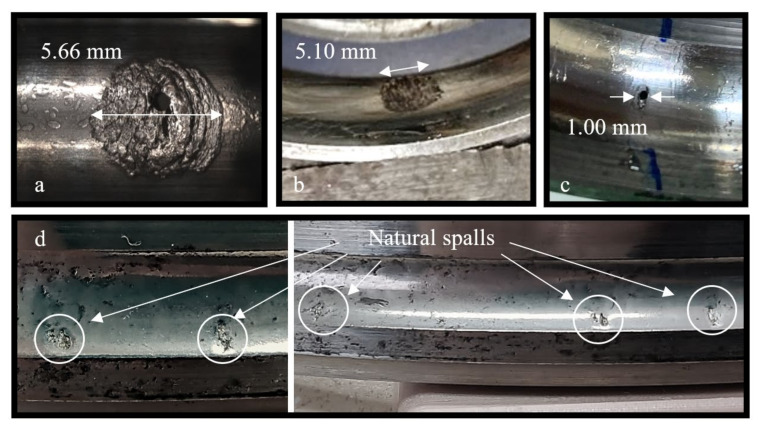
Pictures of spalls in three different experiments: (**a**,**b**) Examples of spalls from the endurance test and (**c**) the disassembly and reassembly test. (**d**) Examples of several in-service spalls that appeared in a Sikorsky CH-53 helicopter swashplate bearing.

The tested bearings were deep groove bearings of type 6206 ETN9, manufactured by SKF from ASTM 52100 steel, with a polymer cage (Emcol, Ashdod, Israel). The polymer cage enables disassembling and reassembling of the bearing without impairing its proper operation. The test rig consisted of a shaft, two support bearings, the tested bearing, and a pneumatic piston for applying radial (vertical) load on the bearing. A schematic drawing and a picture of the test rig can be seen in Figure 7. The rotation speed of the tested bearing was 35 Hz, and it was loaded by the pneumatic piston that exerted a force of 2200 N during all experiments. In order to accelerate the defect initiation process, a small bore was seeded into the outer raceway of the bearings using electrical discharge machining (EDM). The bore was drilled through the thickness and in the center of the outer raceway (Figure 8a), with a needle of diameter 0.75 mm. Figure 9a–c presents three examples of spalls in the final stages of the endurance experiments. To avoid the side effects of the initial bore, the smallest tested spall was twice as the initial bore. To ensure that, several spalls with different sizes were compared. Similar results, i.e., crack’s location and behavior, are shown in each one of the tested spalls. For each of the experiments, accelerometers (Dytran 3263A2 Triaxial sensors, Chatsworth, CA, USA), and Fiber Bragg Grating (FBG) sensors (Tel Aviv university, Israel) were connected to the tested bearing house to measure the vibrations and strains during the experiment with sampling rate of 50 kHz and 10 kHz, respectively. The data acquisition module for the accelerometers is NI PXI-449x and for the FBG is the SMART FIBER interrogator, type Aero Mini. In addition, a magnetic pickup sensor and a thermocouple were connected to the test rig to monitor the experiment. To investigate the process of spall propagation, six experiments were conducted. Five of the experiments were endurance tests and the bearings were disassembled only when failure was recognized. In the sixth experiment, the tested bearing was disassembled, inspected, and reassembled five times with an interval of five operating hours between each disassembly. Between every disassembly and reassembly, optical and scanning electron microscope (SEM) images of the spall were taken. During this sixth experiment, a micro-camera ScoutCam (Omer, Israel) [39] with integrated LEDs was fixed to the tested bearing house to track the defect propagation at any given moment. At first, the bearing ran for approximately 300 h with lubrication until cracks appeared in the artificial defect area. Then, in order to achieve better visibility with the micro-camera, the experiment continued without lubrication for a short period of five hours, without causing thermal failures. At the end of the experiment, the spall area was imaged by SEM and scanned by micro-CT. In addition to the six lab experiments, several in-service spalls that appeared in a Sikorsky CH-53 helicopter (Stratford, CT, USA) swashplate bearing were examined (Figure 9d). 

### 3.2. Experimental Results

The repeated loading of the REs on the defect edge led to the formation of surface cracks, propagating in the deformed surface regions at the trailing edge of the bore [40]. Based on the simulation results in Section 2, sub-surface cracks were expected to appear on the trailing edge, in addition to the surface cracks. In this section, the findings from the experiments are presented.

#### 3.2.1. Disassembly and Reassembly Test Findings

**Figure 10:**
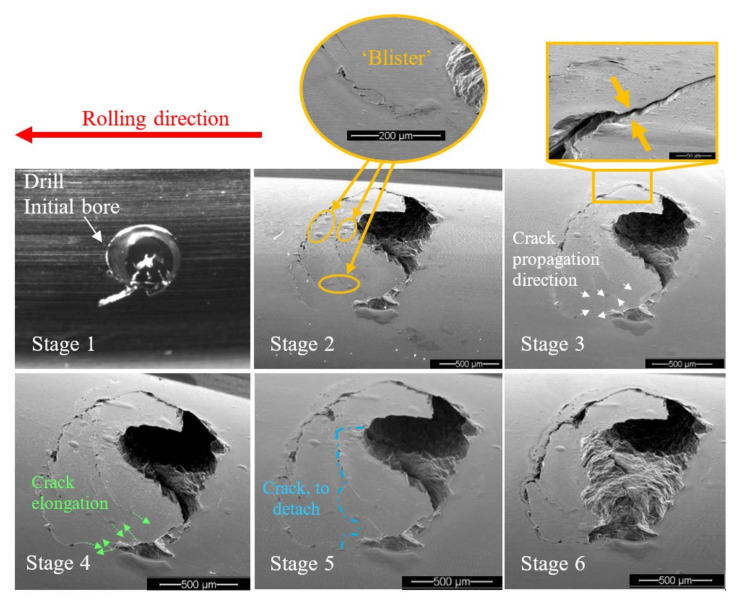
Images from the disassembly and reassembly test. The net crack propagation direction is marked by white arrows in Stage 3. The crack elongation from Stage 3 to Stage 4 is marked by green arrows in Stage 4. “Blisters” at the tip of cracks appear to delay the crack propagation on the surface. The blue dashed line represents the fragment that is about to detach from the raceway between Stages 5 and 6.

Two types of crack propagation can be inferred from the observation of the disassembled and reassembled bearing: (1) propagation of the net cracks on the trailing edge surface (Figure 10, Stage 3—marked by white arrows and Stage 4—marked by green arrows) and (2) propagation of cracks into the sub-surface of the trailing edge (Figure 8 and Figure 10). The cracks of the first type are apparently responsible for the fragment shape, and the second type for the fragment release from the raceway. 

After a fragment is released from the raceway, it is rolled onto the surface and overridden by the REs, creating a blister-shape in the vicinity of the spall. These blisters caused plastic deformation and seemed to delay the crack propagation on the surface. This phenomenon can be observed in Figure 10 by comparing the extent of cracks in Stage 2 to Stage 3. 

The accumulated plastic deformation at the spall edge, as a result of the RE–spall impact, generated residual stresses, that were both compressive and tensile [34,35]. The appearance of a crack in the plastically deformed area released the residual stress and created a “step” between the fragment and the raceway surface, as observed in Figure 10, Stage 3. 

**Figure 11:**
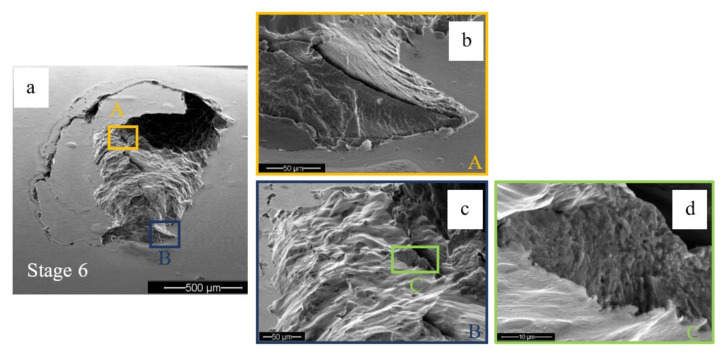
Two suspected areas, “A” and “B” with periodic marks were observed. (**a**) The final stage of the disassembly and reassembly test. (**b**) Close-up image of the periodic marks in area “A”, (**c**) Close-up of area “B”, and (**d**) of periodic marks in area “C”.

The crack propagation in the vicinity of a spall is a result of fatigue. Suspected periodic marks were observed by SEM (Figure 11b,d) although it is difficult to decisively determine that they are fatigue striations since the fragment was pressed on the new surface by the REs before it was released from the raceway, and the REs override onto the new surface of the raceway after the release of fragments and it erodes the spall’s new surface. 

#### 3.2.2. Endurance Test Findings

**Figure 12:**
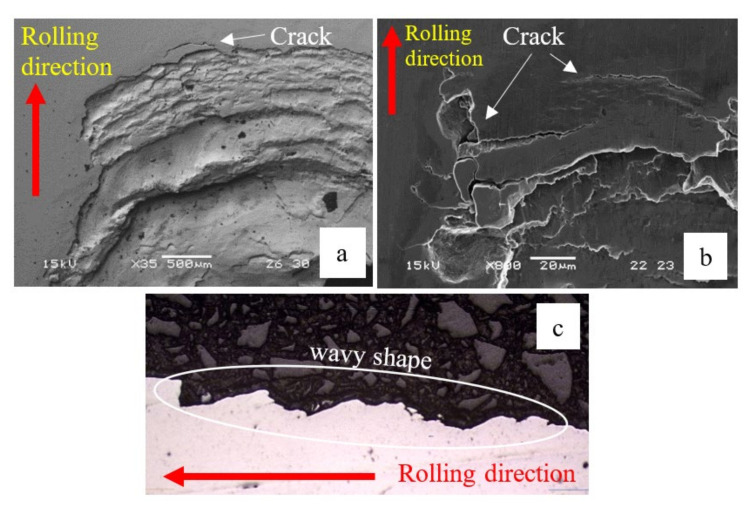
(**a**,**b**) SEM images of the trailing edge of the spall from the endurance tests. Cracks appeared in front of the spall, which eventually cause the spall to propagate. (**c**) Cross-section of the spall (parallel to the rolling direction). The spall bottom has a wavy shaped ”inclined steps” pattern.

**Figure 13:**
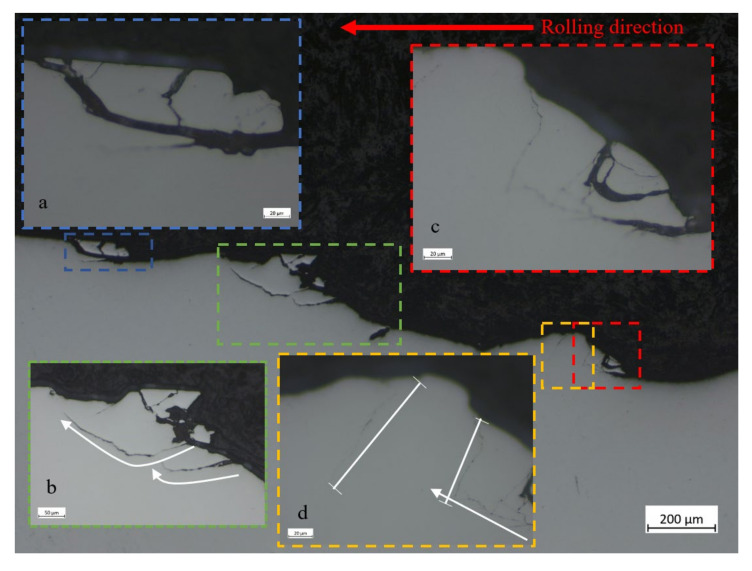
Microscope images of the bottom of spalls. Several fragments that were about to disconnect from the raceway can be observed. (**a**) Fragment release from the edge of the spall. (**b**) Mechanism of single sub-surface crack that form a fragment. (**c**,**d**) mechanism of two cracks that form a fragment. The white arrows and lines represent the direction of cracks propagation and arrested cracks respectively.

Cross-sections parallel to the rolling direction are presented in Figure 12 and Figure 13. They show a wavy “inclined steps” pattern, at the bottom of the spall. Surface and sub-surface cracks can be clearly seen in Figure 12a,b and Figure 13.

**Figure 14:**
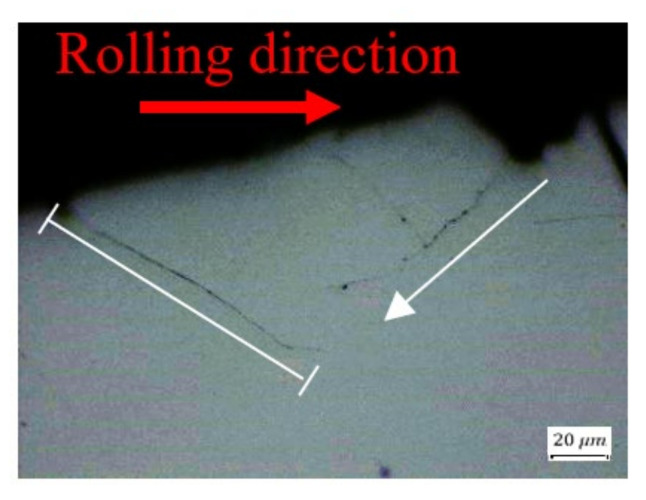
Microscope image of crack propagation within the spall. The white arrow and line represent the direction of crack propagation and arrested crack respectively.

Figure 13 shows several fragments at the bottom of the spall that are about to be disconnected from the raceway. From these fragments, we can learn about the spall evolution process. Whether surface or sub-surface cracks are initiated first cannot be decisively determined from Figure 13a. However, in Figure 14, it seems that the sub-surface cracks already had propagated horizontally before the trailing edge cracks propagated vertically. Apparently, the sub-surface cracks reach a certain length and cease until the trailing edge cracks reach them, which eventually causes a fragment release. Another possibility is that the cracks were initiated simultaneously, but the sub-surface cracks propagated faster than the trailing edge cracks. Figure 13b illustrates a second way to form a fragment, by a single sub-surface crack. The crack propagates parallel to the surface until, at a certain point, the direction of propagation changes and the crack reaches the surface, such that it releases a fragment. The third way to form a fragment is the reverse of the first way, as may be deduced from Figure 13c,d. Once the vertical trailing edge cracks reach a certain length, they cease to propagate. Then, the sub-surface cracks approach the trailing edge cracks, and eventually cause the fragment release. It may also happen that the two types of cracks are initiated simultaneously but, as mentioned, the trailing edge crack propagate faster than the sub-surface cracks.

**Figure 15:**
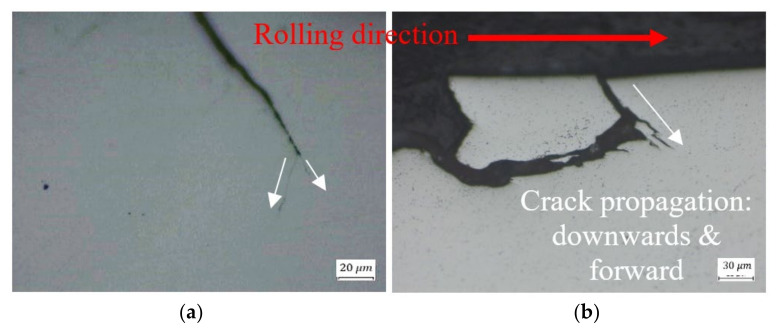
Crack propagation (**a**) into the material depth, and (**b**) towards the rolling direction. The white arrows represent the direction of cracks propagation.

Most of the examined cracks are branched, propagating even in unexpected directions, such as into the material depth (Figure 13c and Figure 15a) or towards the rolling direction in the trailing edge (Figure 15b). In Figure 15b, the crack propagated downwards and forward, and moved away from the sub-surface crack. The cracks which control the spall propagation are those that reach the trailing edge surface by one-crack or two-crack mechanisms, and propagate to the trailing edge surface (as a net of cracks) until the fragment is released. 

**Figure 16:**
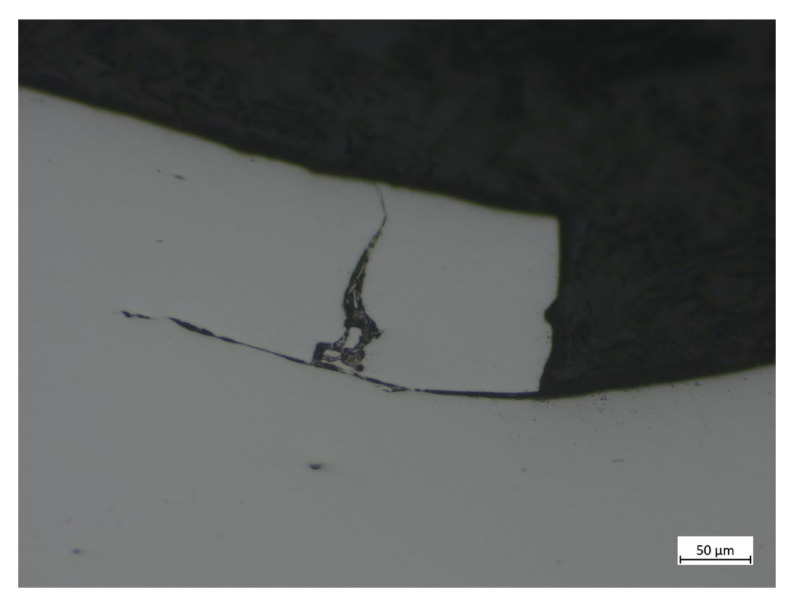
Spall propagation in the axial direction.

Figure 16 illustrates a sub-surface crack in an axial section, while the edge surface crack seems to appear later and to cause fragment release at the loading stage.

#### 3.2.3. In-Service Spall Findings

**Figure 17:**
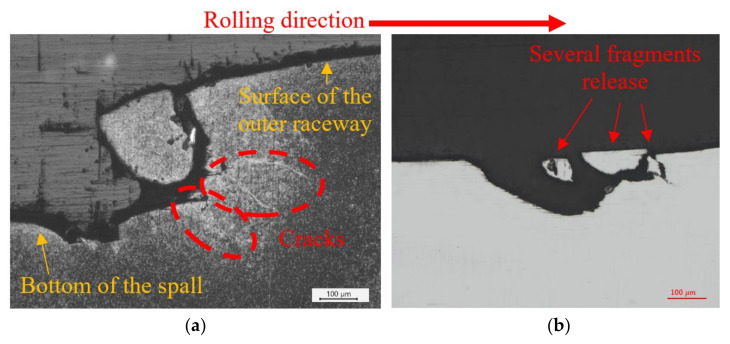
Natural in-service spalls that appeared in a Sikorsky CH-53 helicopter swashplate bearing. (**a**) Appearance of sub-surface cracks and fragment release from the trailing edge of the spall. (**b**) Several fragments released from the trailing edge of the spall.

**Figure 18:**
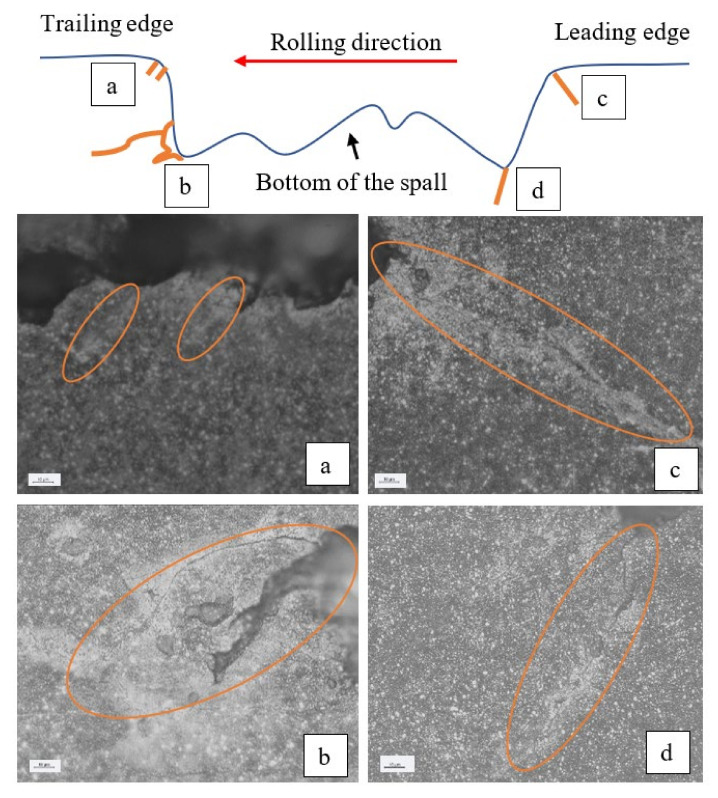
SEM images from the spall: (**a**) The top of the trailing edge of the spall, (**b**) the bottom of the trailing edge, (**c**) the top of the leading edge of the spall, and (**d**) the bottom of the leading edge. The Orange ovals mark the cracks.

Similar observations can be seen in the in-service spalls that appeared in a Sikorsky CH-53 helicopter swashplate bearing. Figure 17a illustrates the formation of sub-surface crack in the trailing edge and a large fragment that was about to be disconnected from the raceway. Figure 17b illustrates several small fragments that were about to be disconnected from the raceway. A schematic description of the spall and four areas of interest are presented in Figure 18a–d. The surface and sub-surface cracks in the trailing edge are presented in Figure 18a,b. Additional cracks are also observed in the leading edge, Figure 18c,d, which indicates propagation of the spall in the opposite direction.

**Figure 19:**
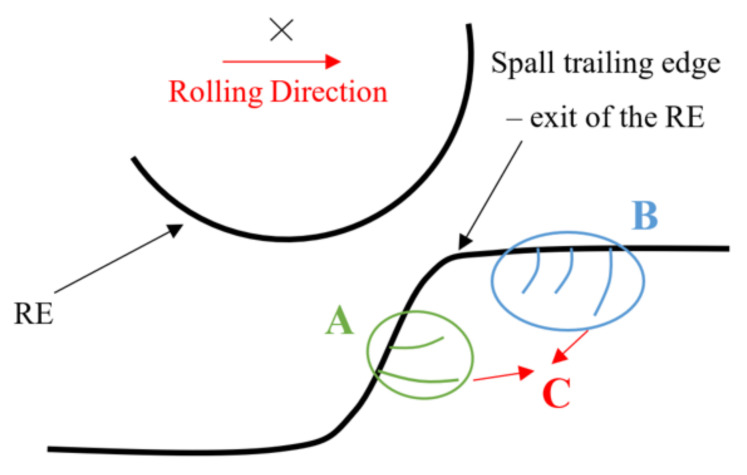
Schematic description of the propagation stages of a spall. “A” is a propagation stage where sub-surface cracks appear underneath the spall trailing edge. In Stage “B”, cracks appear in front of the trailing edge of the spall, and in Stage “C”, the cracks propagate and coalesce until a fragment is released from the raceway.

Based on the experimental observations, spall propagation can be envisioned as a three-stage process, schematically illustrated in Figure 19. In Stage “A”, sub-surface cracks appear underneath the spall trailing edge. In Stage “B”, cracks appear in front of the trailing edge of the spall, and in Stage “C”, the cracks propagate until a fragment is released from the raceway. It is noteworthy that there is no clear experimental evidence, based on our experimental results and on the literature, as to which stage starts earlier, cracks of type “A” or “B”. 

**Figure 20:**
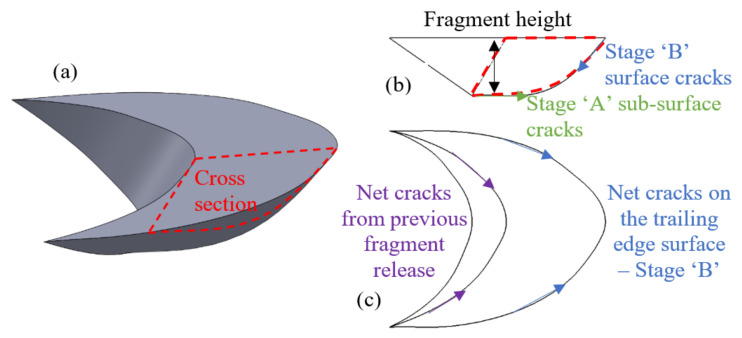
Schematic description of the fragments. (**a**) Isometric view. (**b**) Cross section view. (**c**) Top view.

The estimated shape of the fragment is illustrated in Figure 20. The net cracks evolve on top of the trailing edge and downwards into the sub-surface (Stage “B”), forming the fragment shape (Figure 20b,c). The shape of the fragment also depends on the previous fragments released by net cracks. The sub-surface cracks (Stage “A”) approach the trailing edge cracks and eventually cause the fragment release (Figure 20b). The height of the fragment is less than the location of the maximum shear stress in the material, which gives rise to the appearance of a microcrack in the spall initiation process [34,41]. 

## 4. Conclusions

A combined model of a rigid body dynamic model and a finite element model that simulates the RE–spall edge interaction was used to understand the material behavior (displacements, strains, and stresses) that create an environment for crack formation and propagation. In addition, experiments of bearings with spalls of different sizes that propagate naturally in the laboratory and in the field were conducted and analyzed. Based on the simulation and experiment results, the following conclusions may be made:

- Based on the combined model, two areas with tension stresses were detected in front of the trailing edge and on the free edge of the spall.
- A three-stage mechanism controlling the spall propagation was identified in all the tested bearings both in the laboratory and field. In Stage A, sub-surface cracks appear underneath the spall trailing edge. In Stage B, cracks appear in front of the trailing edge of the spall, and in Stage C, the cracks propagate until a fragment is released from the raceway.
- Several surface and sub-surface cracks were formed simultaneously. The first two cracks—a surface crack and sub-surface crack—that connect to each other, or the first crack that reaches to the surface, release the fragment and cause different fragment sizes and shapes.
- The surface cracks forming the fragment shape and the sub-surface cracks approach the trailing edge cracks and eventually cause the fragment release. The height of the fragment is less than the location of the maximum orthogonal shear stress in the material which corresponds to the appearance of a microcrack in the spall initiation process.
- Several blisters were observed in front of the spall edge and cause plastic deformation which delay the crack propagation on the surface. In addition, the release of residual stresses as a result of crack formation on the surface creates a “step” between the fragment and the raceway surface which later can change the crack propagation direction.
- The fragment release from the raceway creates a wavy shaped “inclined steps” pattern at the bottom of the spall.
- Most of the examined cracks in the experiments were branched, propagating even in unexpected directions, such as into the material depth or toward the rolling direction in the trailing edge.

Future work will investigate the propagation of the spall into the leading and the axial direction. The differences between the various mechanisms will be examined.

## Figures and Tables

**Table 1:** FE model parameters.

Parameters	Value	Units
Spall depth—d	0.125	mm
Spall size—$\Delta_{s}$	2.6	mm
Impact location $\left(x_{imp},y_{imp}\right)$	$\left(1.3,2.1\cdot 10^{-3}\right)$	mm
Normal contact load—$F_{n}$	1900	N
Ball radius—$R_{RE}$	6.4	mm

## Data Availability

Not applicable.
